# Indomethacin Inhibits Cancer Cell Migration via Attenuation of Cellular Calcium Mobilization

**DOI:** 10.3390/molecules18066584

**Published:** 2013-06-04

**Authors:** Yuh-Cherng Guo, Che-Mai Chang, Wen-Li Hsu, Siou-Jin Chiu, Yao-Ting Tsai, Yii-Her Chou, Ming-Feng Hou, Jaw-Yan Wang, Mei-Hsien Lee, Ke-Li Tsai, Wei-Chiao Chang

**Affiliations:** 1 Department of Neurology, Changhua Christian Hospital, Changhua 500, Taiwan; 2 Department of Clinical Pharmacy, School of Pharmacy, Taipei Medical University, Taipei 11031, Taiwan; 3 Master Program for Clinical Pharmacogenomics and Pharmacoproteomics, School of Pharmacy, Taipei Medical University, Taipei 11031, Taiwan; 4 Department of Physiology, College of Medicine, Kaohsiung Medical University, Kaohsiung 807, Taiwan; 5 Department of Urology, College of Medicine, Kaohsiung Medical University Hospital, Kaohsiung Medical University, Kaohsiung 807, Taiwan; 6 Cancer Center, Kaohsiung Medical University Hospital, Kaohsiung Medical University, Kaohsiung 807, Taiwan; 7 Division of Gastroenterologic and General Surgery, Department of Surgery, Kaohsiung Medical University Hospital, Kaohsiung Medical University, Kaohsiung 807, Taiwan; 8 Graduate Institute of Clinical Medicine, College of Medicine, Kaohsiung Medical University, Kaohsiung 807, Taiwan; 9 Graduate Institute of Pharmacognosy, College of Pharmacy, Taipei Medical University, Taipei 11031, Taiwan; 10 Department of Pharmacy, Taipei Medical University-Wanfang Hospital, Taipei 11031, Taiwan

**Keywords:** indomethacin, EGF, COX-2, cell migration

## Abstract

Non-steroidal anti-inflammatory drugs (NSAIDs) were shown to reduce the risk of colorectal cancer recurrence and are widely used to modulate inflammatory responses. Indomethacin is an NSAID. Herein, we reported that indomethacin can suppress cancer cell migration through its influence on the focal complexes formation. Furthermore, endothelial growth factor (EGF)-mediated Ca^2+^ influx was attenuated by indomethacin in a dose dependent manner. Our results identified a new mechanism of action for indomethacin: inhibition of calcium influx that is a key determinant of cancer cell migration.

## 1. Introduction

There is growing interest in understanding the mechanisms regulating the antiproliferative effects of non-steroidal anti-inflammatory drugs (NSAIDs). Studies obtained from* in vitro* experiments indicate that NSAIDs can block cell proliferation, resulting in inhibition of cell growth [1,2]. Indomethacin, one of the most common NSAIDs, possesses anti-inflammatory, analgesic, and antipyretic properties by non-selectively inhibiting both cyclooxygenase (COX)-1 and COX-2 [3]. Eli* et al.* reported that a low dose of indomethacin causes acceleration of apoptosis and inhibition of cell proliferation [4].

NSAIDs inhibit synthesis of prostaglandins from arachidonic acid by the COX enzymes [5]. COX-1 is constitutively expressed and is required for physiological processes such as maintenance of the gastrointestinal mucosa and vascular homeostasis, whereas COX-2 is an inducible enzyme that has been linked to inflammatory reactions and cytokine release [6]. The *COX-2* gene is overexpressed in human colon cancer, and the high level of COX-2 expression is correlated with mutagenesis and angiogenesis [7].

Although several studies have demonstrated the protective effects of NSAIDs in tumor development and progression [8,9], the molecular mechanism of how NSAIDs are involved in inhibiting cancer cell focal adhesion and migration is still unclear. Vinculin is a major component of focal adhesions that can be detected by immunostaining of vinculin. Vinculin is a cytoskeletal protein that localized in integrin-mediated cell-matrix adhesions and cadherin-mediated cell-cell junctions [10]. Previous studies reported that vinculin-mediated focal adhesion is a calcium dependent process that is involved in cancer cell migration [10]. Blocking store-operated Ca^2+^ influx slows down focal adhesion turnover that resulted in stronger adherence [10].

The aim of this study was to clarify the molecular mechanism of how indomethacin influences cell migration in cancer cells. We hypothesized that indomethacin may interfere with calcium-dependent pathways, which in turn, contribute to blocking cancer cell migration. To test this hypothesis, we examined interactions among cell migration, Ca^2+^ mobilization, and vinculin (focal complexes) in cancer cells. Our results revealed that indomethacin may indeed inhibit cancer cell migration by influencing calcium mobilization and focal complex formation.

## 2. Results and Discussion

### 2.1. Effects of Indomethacin on COX-2 Gene Expression

**Figure 1:**
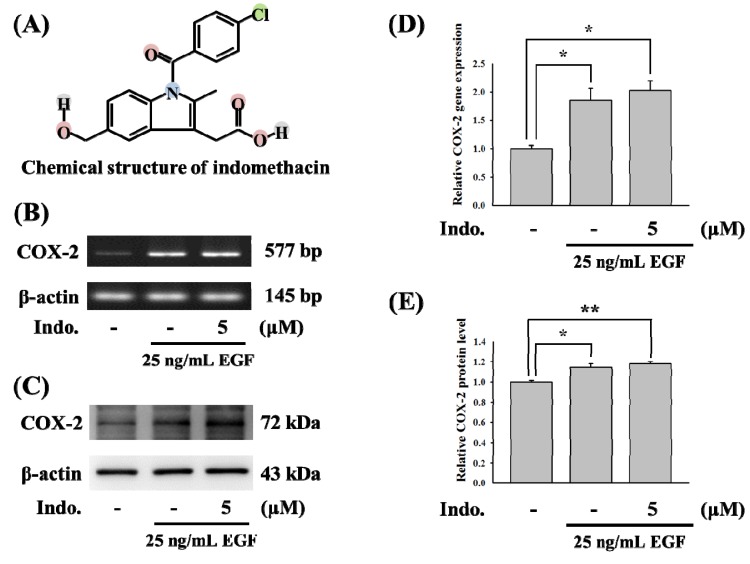
Effects of indomethacin on EGF-mediated COX-2 gene. (**A**) The chemical mRNA level of *COX-2*. RNAs were isolated from A431 cells, and expression of *COX-2* gene was measured by RT-PCR. (**C**) Effects of indomethacin with 25 ng/mL EGF induction on COX-2 expression in A431 cell line. Cell lysates were prepared for detecting the protein level of COX-2 and β-actin by western blotting. (**D**,**E**) Relative quantification of COX-2 mRNA levels and protein expression were conducted using ImageJ software (* *p* < 0.05).

The chemical structure of indomethacin is shown in Figure 1A. As is known, indomethacin is a well-known NSAID that targets COX. To test the effects of indomethacin on *COX-*2 gene expression, we first used EGF-mediated *COX-2* gene expression as a model. As shown in Figure 1B, 5 μM indomethacin cannot inhibit EGF-induced *COX-2* gene activation. The COX-2 protein level was not influenced by 5 μM indomethacin (Figure 1C). Statistical analysis of the results from COX-2 gene and protein expression were shown in Figure 1D,E.

### 2.2. Indomethacin Inhibits Cancer Cell Migration

**Figure 2:**
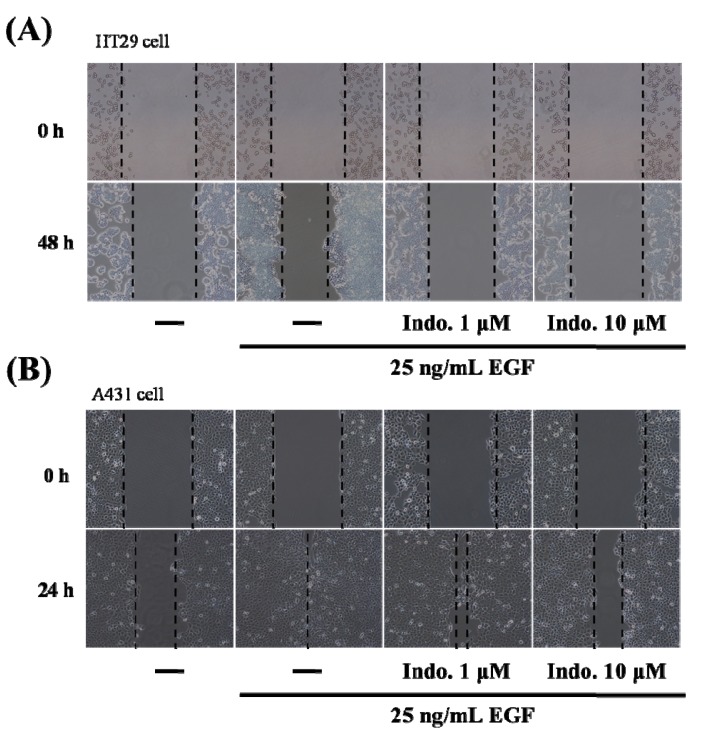
Effects of indomethacin on cell migration. (**A**) The cell migration of HT29 cells after 48-hour treatment with indomethacin and EGF was measured by wound-healing assay. Cells were pretreated with 1 or 10 uM of indomethacin and then stimulated with 25 ng/mL EGF. (**B**) The cell migration of A431 cells after 24-h treatment with indomethacin and EGF was measured by wound-healing assay. Cells were pretreated with 1 or 10 uM of indomethacin and then stimulated with 25 ng/mL EGF.

Cell migration is a key step in initiating the tumor metastatic cascade. Wound-healing assays are widely used to provide information on cell migration. In our study, HT29 colon cancer cells and A431 EGFR-positive cancer cells were treated with different concentrations of indomethacin combined with the EGF, and wound closure was analyzed. Our results revealed the inhibitory effects of indomethacin on the migration of HT29 cells (Figure 2A). After treatment of A431 cells with only the EGF, the wound closed in 24 h, but the wound was still open after treatment with indomethacin for 24 h (Figure 2B). Furthermore, the extent of wound closure was correlated with the concentration of indomethacin. These results indicated that indomethacin can inhibit cancer cell migration.

### 2.3. Effects of Indomethacin on Focal Complexes Formation

**Figure 3:**
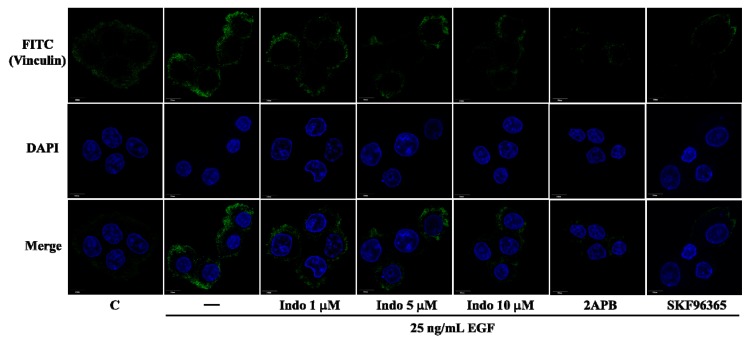
Effects of indomethacin on vinculin expression. The immunofluorescence imaging of vinculin in A431. Cells were pretreated with 1, 5 and 10 μM indomethacin or SOCE inhibitors, 100 μM 2APB and 20 μM SKF96365 for 30 min, then stimulated with 25 ng/mL EGF for 3 h. Vinculin (green) and nucleus (blue) are stained by FITC-conjugated antibody and DAPI, respectively.

Vinculin, a cytoskeletal protein, is localized in integrin-mediated cell-matrix adhesions [10]. Vinculin involves in focal complexes formation and cancer cell migration [11]. Previous studies have indicated that EGF can activate store-operated Ca^2+^ influx [12] and store-operated calcium entry is essential in focal adhesions [13,14]. To further clarify how indomethacin inhibited cell migration, the imaging from immunofluorescence assay was employed to detect the focal complexes formation. As showed in Figure 3, EGF-mediated focal complexes formation was detected at the cell membrane. Importantly, pretreatment with store-operated Ca^2+^ entry (SOCE) inhibitors, SKF96365 and 2APB, blocked the focal complexes formation (Figure 3). The results from SOCE inhibitors were similar to that from indomethacin.

### 2.4. Effects of Indomethacin on EGF-Medicated Ca^2+^ Signaling

**Figure 4:**
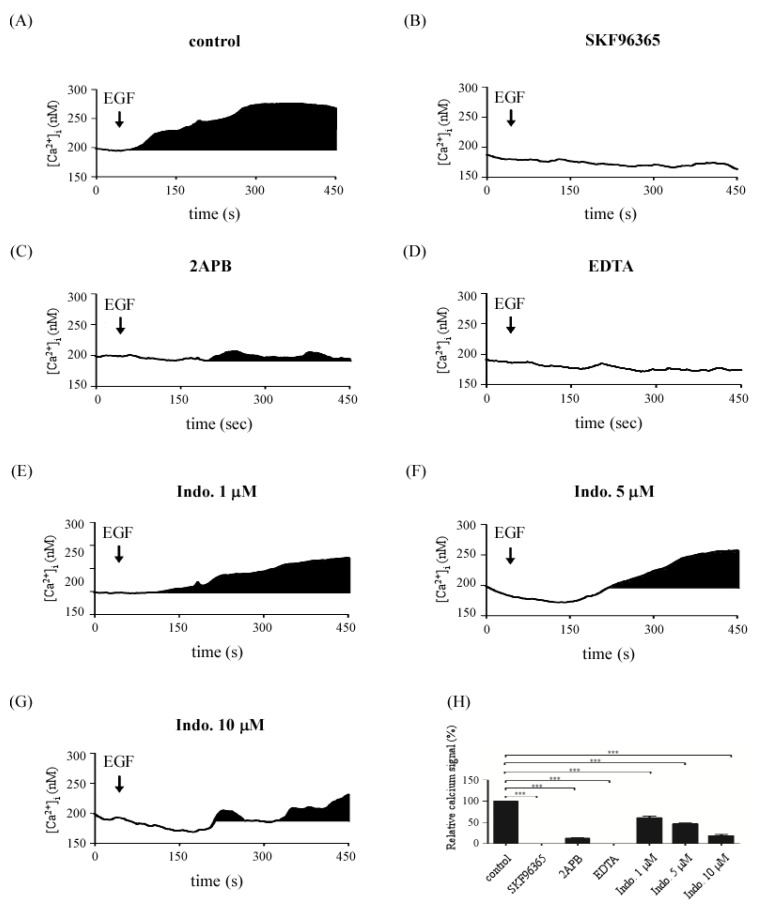
Effects of indomethacin on EGF–mediated Ca^2+^ influx. Time course of calcium signals following exposure to 25 ng/mL EGF. The Ca^2+^ influx signals were evoked by EGF stimulation (**A**), and with the pretreatment of SKF96365 (**B**), 2APB (**C**), 1 mM EDTA (**D**), 1 μM (**E**), 5 μM (**F**) or 10 μM (**G**) indomethacin. The cells were loaded with Fluo-4-AM for Ca^2+^ detection and the Ca^2+^ response was due to a difference in the time constant utilized for averaging the signal. (**H**) The Ca^2+^ signals were estimated by calculating the black areas under the Ca^2+^ curve (*** *p* < 0.005).

Results from Figure 3 indicated that both SOCE inhibitors and indomethacin can block focal complex formation. Calcium is a key signal for focal complex formation. Therefore, we further investigated the effects of indomethacin on EGF-medicated store-operated Ca^2+^ influx. Cells were pretreated with SKF96365, 2APB and the different concentration of indomethacin for 30 min and then calcium signals were detected. As shown in Figure 4, EGF-induced store-operated Ca^2+^ influx (Figure 4A) was blocked by SKF96365 (Figure 4B,H) and 2APB (Figure 4C,H), respectively. 1 mM EDTA, as a positive control, was applied to block EGF-induced Ca^2+^ influx (Figure 4D). Importantly, indomethacin repressed EGF-induced store-operated Ca^2+^ influx in a dose dependent manner (Figure 4E–H). These results implied that indomethacin blocked cell migration (focal complexes formation) via attenuation of calcium mobilization evoked by EGF.

### 2.5. Effects of Indomethacin on EGF-Medicated EGFR Phosphorylation

**Figure 5:**
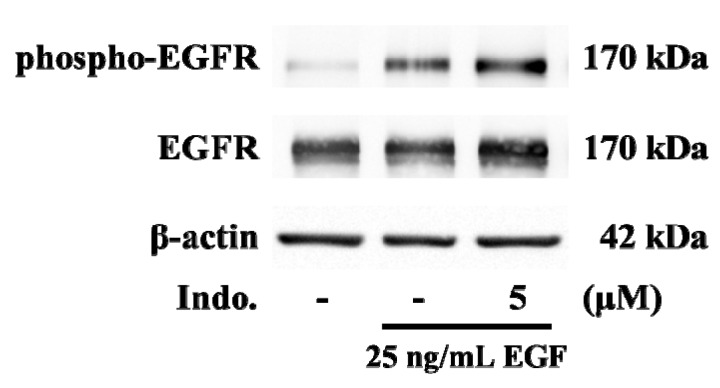
Effects of indomethacin on EGF-mediated phosphorylation of EGFR. Cells were pretreated with 5 μM indomethacin for 30 min and then stimulated with 25 ng/mL EGF for 30 min. The cell lysate was isolated from A431 cells and was analyzed by western blotting for detecting EGFR and phosphor-EGFR^(Y1173)^ expression.

Next, we investigated whether any proteins in the upstream of calcium signaling are involved in the inhibitory effects of *indomethacin*. It has been reported that the Tyr residue (position, 1173) in EGFR can be phosphorylated by EGF [15]. Therefore, we further checked for the phosphorylation of EGFR by western blotting. Immunoblotting analysis showed that the phospho-EGFR^Y1173^ level was induced at 30 min after stimulation with EGF (Figure 5). However, EGF-induced phosphorylation of EGFR^Y1173^ was not changed by pretreatment with 5 μM of indomethacin (Figure 5).

### 2.6. Discussion

In human breast cancer, overexpression of COX-2 is involved in tumor cell invasion of blood vessels [16]. Cell-based experiments indicated that the *COX-2* gene is transcriptionally activated by EGF signaling. Both the EGFR and COX-2 are important regulators of tumor invasion and metastasis [17]. In this study, EGF-mediated COX-2 gene expression and cell migration were used as a model to identify the functional effects of indomethacin. To determine whether indomethacin’s effect was linked to COX-2-inhibiting properties or was independent of them, a low dose of indomethacin was used. Consistent with previous studies, our results indicated that 5 μM of indomethacin had no inhibitory effect on COX-2 expression. Importantly, this dose was sufficient to significantly block EGF-mediated cancer cell migration. Even so, we still could not conclude that indomethacin blocks cell migration via a COX-2-independent mechanism. Although neither the *COX-2* gene expression nor protein was inhibited by indomethacin, the enzyme activity of COX-2 might be the main target of indomethacin. More research is needed to determine the correlation between COX-2 activity and the pathogenesis of tumor cells in an* in vivo* animal model.

In non-excitable cells such as T cells, B cells, and cancer cells, the major calcium entry pathway is through store-operated calcium channels (SOCCs) [18]. Calcium entry via SOCCs is necessary for tumor cell migration and metastasis [14]. Calcium mobilization through SOCCs is a major determinant of the expression of the proliferative marker, vinculin, and cell migration [14]. Indeed, it is well established that Ca^2+^-NFAT signaling regulates cell differentiation and development in many different cell types and organ systems. Numerous studies over the last few years documented aberrant NFAT signaling in tumor development and metastasis [19]. In this study, intracellular calcium concentration was reduced by indomethacin. In addition to SOCC, EGF may also up-regulate other types of calcium channel, such as transient receptor potential (TRP) channel. In fact, it has been reported EGF enhances migration of A549 lung cancer cells via TRPM7 channel, and arachidonic acid can act as a modulator of TRPM5 channels [20,21]. Further investigations to test the effects of indomethacin on calcium currents are necessary.

Numerous studies reported that store-operated calcium channel appears to be an important mechanism involved in cancer cell migration and inflammatory gene expression [14,22,23]. Kokoska* et al.* indicated that NSAIDs such as indomethacin, ibuprofen, and aspirin may influence EGF-mediated calcium signaling by altering intracellular calcium mobilization [24,25]. Carrasco-Pozo* et al.* further indicated that indomethacin stimulates endoplasmic reticulum calcium mobilization and the subsequent entry of calcium into the mitochondria [26]. Consistent with this, we also observed a small increased calcium signals evoked by indomethacin (Figure 4C,G). Because our findings are in good agreement with the detailed study by Kokoska* et al.* and Carrasco-Pozo* et al.*, we speculate that indomethacin inhibited bulk cytosolic calcium concentration via the endoplasmic reticulum calcium mobilization and mitochondrial calcium buffering mechanisms.

Our study has limitations. First, although both 2-APB and SKF96365 have been widely used to block store-operated calcium channel, the inhibitory effects of these compounds are not specific. Extrapolation of conclusions from experiments on pharmacological inhibitors (2APB and SKF96365) to the involvement of store-operated calcium channels requires validation of the siRNA screen. Second, we observed that 5 μM indomethacin inhibited EGF-mediated calcium signals but not COX-2 gene expression. To determine whether the effects of indomethacin are COX-2 independent, it is necessary to determine the inducible COX-2 activity. Third, our results indicated that phosphorylation of EGFR^Y1173^ cannot be blocked by indomethacin. C-terminal phosphorylation sites of EGFR includes 992, 1068, 1148 and 1173 tyrosine residues that can serve as specific docking sites for the Src homology domain 2 (SH2) or phosphotyrosine binding (PTB) domain of adaptor protein [27,28]. Further studies are necessary to understand whether other EGFR phosphorylation sites are involved in the inhibitory effects of indomethacin.

**Figure 6:**
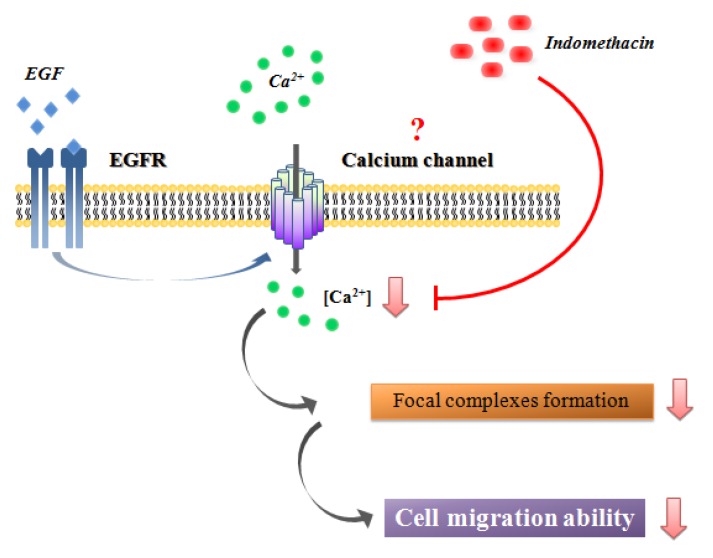
Schematic representation of the mechanisms of indomethacin in EGF-mediated COX-2 gene expression.

In conclusion, we first describe a novel mechanism of how indomethacin inhibits cell migration. Our findings highlight how indomethacin can influence calcium influx and further suppress cell migration by decreasing focal complexes (Figure 6). In consequence, indomethacin might be a potential inhibitor of calcium channels.

## 3. Experimental

### 3.1. Cell Culture

Human epidermoid carcinoma A431 cells were bought from ATCC. Cells were cultured (37 °C, 5% CO_2_) in Dulbecco’s modified Eagle’s medium (DMEM) (Invitrogen Corp., Carlsbad, CA, USA) with 10% fetal bovine serum and 1% penicillin-streptomycin. In this series of experiments, cells were treated with 25 ng/mL EGF in serum-free DMEM medium. For Ca^2+^ imaging experiments, cells were prepared onto glass coverslips and used 24–48 h after plating.

### 3.2. Calcium Imaging

Ca^2+^ influx was detected with stimulation by EGF (Sigma-Aldrich, St. Louis, MO, USA). Before the experiments, the cells were stained with 1 μM Fluo-4-AM (Molecular Probes, Eugene, OR, USA) at 37 °C for 20 min and then washed with BSS buffer (5.4 mM KCl, 5.5 mM D-glucose, 1 mM MgSO4, 130 mM NaCl, 20 mM Hepes pH 7.4, and 2 mM CaCl2). Ca^2+^ signals were estimated based on the ratio of fluorescence intensities emitted upon excitation with consecutive 3-second pulses of 488-nm light at a resolution of 1376 × 1038 pixels using an Olympus Cell^R IX81 fluorescence microscope (Olympus, Suite A Hicksville, NY, USA) equipped with an MT 20 illumination system (Olympus) and UPLanApo 10× objective lens. Intracellular Ca^2+^ concentration was estimated based on calibration curves as follows. A Ca^2+^ calibration curve was created using a Ca^2+^ Calibration Buffer kit (Molecular Probes). Intracellular Ca^2+^ ([Ca^2+^]_i_) was calculated from Fluo-4 excited at 488 nm and imaged using an Olympus Cell^R IX81 fluorescence microscope and UPLanApo 10× objective lens at 20 °C. Fluo-4 signals were calibrated by measuring the fluorescence intensity from microcuvettes containing 10 mM K2EGTA (pH 7.20) buffered to various [Ca^2+^] levels. Ca^2+^ concentration was calculated using the following formula: [Ca^2+^]_I_ = KD × (F − F_min_/F_max_ − F). Plotting the fluorescence intensity* versus* [Ca^2+^] yielded the calibration curve with the formula: [Ca^2+^]_I_ = KD × (F − Fmin/Fmax − F), where KD = 150.5 nM, F = Fluo-4 intensity, F_max_ = 640, and F_min_ = 21.7 for Fluo-4 [29].

### 3.3. Reverse Transcriptase PCR

Total RNA was extracted from A431 cells by Trizol (Invitrogen Corp.). A reverse transcriptase reaction was performed on 1 µg of extracted total RNA using reverse transcriptase reaction Kit (Invitrogen Corp.) according to the manufacturer’s instructions. Following cDNA synthesis, gene-specific primers to *COX-2* (577 bps) and *β-actin* (145 bps) were designed using NCBI Primer-BLAST. Primer sequences were as following: COX-2 (sense primer: AGACAGCGTAAACTGCGCCTTT; antisense primer:CAGCAATTTGCCTGGTGAATGATTC); β-actin (sense primer: ATCTCCTTCTGCATCCTGTCGGCAAT; antisense primer: CATGGAGTCCTGGCATCCACGAAAC). Relative quantification of mRNA levels was conducted using Image J software.

### 3.4. Western Blotting

Total cell lysates (20 µg) were analyzed by SDS-PAGE on 12.5% or 10% gel. After electro-blotting to nitrocellulose membrane, membranes were blocked with 5% nonfat dry milk in 0.1% PBST buffer for COX-2 and β-actin or TBST buffer for EGFR, phosphor-EGFR and β-actin for 1 h at room temperature. Membranes were washed with 0.1% PBST or TBST three times and then incubated with primary antibodies overnight at 4 °C. Antibodies were obtained from the following sources: COX-2 from Santa Cruz Biotechnology (Santa Cruz, CA, USA), β-actin from Amersham Biosciences (Piscataway, NJ, USA), and EGFR and EGFR Phospho (pY1173) from Epitomics (Cambridge, MA, USA). COX-2 antibody was used at a 1:3000 dilution, β-actin was used at a 1:10000 dilution, and EGFR as well as phosphor-EGFR were both used at 1:1000 dilution. Then the membranes were washed with 0.1% PBST or TBST three times and incubated with a 1:6000 dilution of peroxidase-linked anti-mouse and 1:3000 dilution of anti-rabbit IgG (Amersham Biosciences) for 1 h at room temperature. After washing with 0.1% PBST or TBST, the bands were detected by an ECL-plus Western Blot Detection System (Millipore Corp., Bedford, MA, USA). The ImageJ software was used to quantify protein expression levels.

### 3.5. Wound Healing Assay

Culture inserts (ibidi, Munich, Germany) were placed in 6 well culture plates. HT29 and A431 cells were seeded 1 × 10^4^/well in culture inserts. After 16 h, the culture inserts were removed. The cells were washed with PBS and maintained in 1 mL DMEM medium with 10% FBS. Cells were pre-treated with 1 or 10 uM indomethacin for 30 min, and then treated with of 25 ng/mL EGF for 24 h (A431) or 48 h (HT29). The cells were then placed onto an inverted microscope (Nikon Eclipse Ti-U, Tokyo, Japan) for imaging. Random fields in each well were selected for imaging with a Nikon Plan Apo 10X/0.3 objective using a digital sight DS-U2 controlled by NIS-Element D software.

### 3.6. Immunofluorescence

Cells were seeded onto the glass coverslips in 6 well plastic plates to grow for 1–2 days. Before immunostaining, cells were pretreated with or without 1 or 10 uM indomethacin for 30 min, and then stimulated by 25 ng/mL EGF for 3 h. Following the treatment, cells were washed with PBS; then fixed with 4% formaldehyde and permeabilized with 0.1% Triton X-100 in PBS. In between the fixing and permeabilizing steps, cells were washed with 0.2% PBST for 10 min. After blocking cells with 1% BSA, primary antibody was added and cells were incubated at 4 °C overnight and then washed with 0.2% PBST for 10 min. Cells were subsequently incubated with fluorescein isothiocyanate (FITC)-conjugated secondary antibody for 1 h; then with 4',6-diamidino-2-phenylindole (DAPI) for 1 h. 0.2% PBST was used to wash cells 3 times each for 5 min in between staining. Coverslips were inverted and fixed onto glass slides by 50% glycerol. The fluorescence of FITC and DAPI were detected by confocal microscope.

### 3.7. Data Analysis

Statistical analyses were performed using Student’s t-test. A *p*-value less than 0.05 was considered significant and was denoted by *, and *p*-value less than 0.01 was denoted by **.

## 4. Conclusion

Our results identified a new mechanism of action for indomethacin: inhibition of EGF-mediated calcium signals that is a key determinant of cancer cell migration.
